# Adequacy of Venous Thromboembolism Risk Assessment and Prophylaxis After Gastrointestinal Surgery in a Sudanese Teaching Hospital: A Prospective Audit

**DOI:** 10.7759/cureus.73316

**Published:** 2024-11-09

**Authors:** Muaz Hassan, Rami A Adam, Mosab Hussen Mostafa Adam, Rawan Mairghani, Almegdad S Ahmed, Hadeel Abdelseid, Alaa Abdalla, Fatima Mohamedahmed Babiker Mohamed, Ghofran B Yousif, Mohamed A Adam, Hussein A Embarek, Omer H Salim

**Affiliations:** 1 General Surgery, Alnao Teaching Hospital, Khartoum, SDN; 2 General Surgery, Alexandria University, Cairo, EGY; 3 Orthopedics, University of Khartoum, Khartoum, SDN; 4 General Surgery, University of Khartoum, Khartoum, SDN; 5 Microbiolgy and Parasitology, University of Khartoum, Khartoum, SDN; 6 Obstetrics and Gynaecology, University of Khartoum, Khartoum, SDN; 7 Internal Medicine, University of Khartoum, Khartoum, SDN; 8 Internal Medicine, National University Sudan, Khartoum, SDN; 9 Internal Medicine, Ribat University Hospital, Khartoum, SDN; 10 General Surgery, Tripoli University, Tripoli, LBY; 11 General Surgery, University of Ain Shams, Cairo, EGY; 12 Surgery, Dubai Medical College for Girls, Dubai, ARE

**Keywords:** audit, gastrointestinal surgery, low molecular weight heparin, mechanical prophylaxis, post-operative, venous thromboembolism (vte)

## Abstract

Background

Venous thromboembolism (VTE) is a condition that occurs when a blood clot forms in a vein. VTE includes deep vein thrombosis (DVT) and pulmonary embolism (PE). Regular monitoring and risk assessment are crucial for effectively using VTE prevention measures. This study aimed to evaluate the practices related to VTE risk assessment and prophylaxis within our surgical unit in a Sudanese teaching hospital.

Methods

This study was conducted at Alnao teaching hospital and was comprised of two cycles. It examined adult patients who underwent gastrointestinal operations. Data from medical records including age, sex, type of operation, whether VTE and bleeding risk assessments were performed, whether pharmacological or mechanical prophylaxis was administered, and any contraindications to VTE prophylaxis. The practice of VTE risk assessment and prophylaxis prescription was compared to the National Institute for Health and Care Excellence (NICE) guidelines for VTE risk assessment and prophylaxis. Following cycle one, regular educational sessions were conducted for medical staff, emphasizing the need for improved practices in assessing the risk of VTE and prophylaxis prescription.

Results

32 patients in cycle one and 29 patients in cycle two were included. VTE and bleeding risks were assessed in 0/32 (0.00%) of patients in cycle one compared to 29/29 (100%) in cycle two. In cycle one, 0/32 (0.00%) patients were given VTE prophylaxis according to the guidelines. This practice improved to 17/29 (58.6%) in cycle two.

Conclusion

The audit highlights the role of organized methods and education in improving adherence to VTE prophylaxis guidelines. Targeted interventions like educational sessions and risk assessment tools led to significant practice improvements, particularly in low-resource settings. Continuous auditing and training are essential for maintaining and enhancing compliance with VTE prophylaxis standards.

## Introduction

Venous thromboembolism (VTE) occurs when a blood clot forms in the vein. VTE includes deep vein thrombosis (DVT) and pulmonary embolism (PE) [1]. The incidence rates of VTE are significantly higher in high-income countries (0.87 per 1000 person-years) than in upper-middle-income (0.25) and lower-middle/low-income countries (0.06) [2].

VTE is a major cause of morbidity and mortality in hospitalized patients. It is estimated that more than 50,000 patients die from PE each year in the United States [1]. In the majority of these patients, the diagnosis was not suspected before death, highlighting the fact that fatal PE can be the first manifestation of asymptomatic DVT. Unrecognized DVT can also lead to long-term morbidity from post-phlebitic syndrome and may predispose patients to recurrent VTE [3].

Several factors can increase the risk of VTE, including prolonged immobility and certain medical conditions like cancer, diabetes, heart disease, and obesity. The risk of VTE also increases with age, particularly after 40 [1].

Regular monitoring and risk assessment are crucial for effectively using VTE prevention measures. Studies indicate that implementing VTE risk assessments nationwide in the U.S. could prevent over 300,000 hospital-onset VTE cases annually, reduce the rate of complications, and save approximately $1.5 billion [4-6].

 DVT is common in patients undergoing surgery. It is approximately 14% in gynecological surgery, 22% in neurosurgery, 26% in abdominal surgery, and 45%-60% in patients undergoing hip and knee operations without thromboprophylaxis. A thorough history and physical examination should be conducted to evaluate VTE and bleeding risks, categorizing patients as low, moderate, or high-risk [7,8].

Many international guidelines emphasize the importance of using validated tools like the Caprinin and Roger scores to assess and prevent VTE. These guidelines provide clear instructions for evaluating VTE risk and prescribing appropriate prophylaxis such as anticoagulant therapy (e.g., low-molecular-weight heparin (LMWH), unfractionated heparin (UFH), and fondaparinux) or mechanical methods (anti-embolism stockings or intermittent pneumatic compression) [1,8-11].

Risk assessment and prophylaxis of VTE are under-practiced in developing countries [12-14]. This lapse could affect patients' health and increase the financial burden [15]. Furthermore, implementing VTE prophylaxis protocols in low and middle-income countries (LMICs) presents several challenges. A primary issue is limited resources, as many LMICs like Sudan have restricted healthcare budgets that result in shortages of crucial medications and equipment needed for effective VTE prevention, insufficient training for providers, and the absence of standardized protocols [12,13,16,17]. Additionally, there is often a lack of awareness about VTE risks among both healthcare professionals and patients, which leads to underutilization of available prevention strategies [7,18].

Regular auditing is vital for assessing adherence to VTE prophylaxis guidelines and improving patient outcomes. To tackle this critical issue, we conducted a thorough clinical audit to evaluate the practices related to VTE risk assessment and prophylaxis within our surgical unit in a teaching hospital in Sudan.

## Materials and methods

Study area and design

This prospective audit was conducted in the general surgery department (gastrointestinal unit ) at Alnao Teaching Hospital (Khartoum, Sudan). This study followed the classical audit design which involves assessing the current practice, identifying areas for improvement, applying changes, and re-evaluating the practice. The gastrointestinal unit comprises four consultants, two specialists, 13 registrars, 18 medical officers, and 23 house officers. The average of gastrointestinal-related surgeries is 14 operations per month.

Ethical approval

This study was considered a quality project. It was reviewed and approved by the Alnao University Hospital Research Ethics Committee (AUH-REC) and the Surgical Department on 02/07/2022, with ethical approval number AUH0710202SI. Upon data collection, each patient gave verbal consent.

Population and sampling

Total coverage of all adult patients (age >16 years) who underwent elective or emergency gastrointestinal (GI) operations during the study period.

Criteria and standards

The criteria and standards of this audit were obtained from the NICE guidelines for VTE risk assessment and prophylaxis in individuals aged over 16 [8]. It states that:

All surgical patients should have their VTE and bleeding risks assessed (Tables 1, 2); mechanical prophylaxis, either anti-embolism stockings or intermittent pneumatic compression, is started on admission for people undergoing abdominal surgery unless there is a contraindication; when the patient's risk of VTE outweighs the risk of bleeding, pharmacological VTE prophylaxis (either LMWH or fondaparinux sodium) is added to mechanical prophylaxis for a minimum of seven days for those who require it; for patients at high risk of bleeding, mechanical prophylaxis is preferred over medication. This audit used the Caprini scoring system [10] as a tool for VTE risk assessment, as shown in Table 1.

**Table 1:** Capiring scoring system for VTE risk assessment VTE: venous thrombo embolism

Caprini Score	Risk category	Recommended prophylaxis	Duration of chemoprophylaxis
0	Lowest	Early frequent ambulation only, or at the discretion of the surgical team: pneumatic compression devices or graduated compression stockings	During hospitalization
1-2	Low	Pneumatic compression devices ± graduated compression stockings	During hospitalization
3-4	Moderate	Pneumatic compression devices ± graduated compression stockings	During hospitalization
5-8	High	Pneumatic compression devices and low dose heparin or low molecular weight heparin	7–10 days total
≥9	Highest	Pneumatic compression devices and low dose heparin or low molecular weight heparin	30 days total

**Table 2:** Bleeding risk assessment tool according to the NICE guidelines* * Any tick should prompt clinical staff to consider if bleeding risk is sufficient to preclude pharmacological intervention.

Patient related	Tick	Admission related	Tick
Active bleeding		Neurosurgery, spinal surgery or eye surgery	
Acquired bleeding disorders (such as acute liver failure)		Other procedure with high bleeding risk	
Concurrent use of anticoagulants known to increase the risk of bleeding (such as warfarin with INR >2)		Lumbar puncture/epidural/spinal anaesthesia expected within the next 12 hours	
Acute stroke		Lumbar puncture/epidural/spinal anaesthesia within the previous 4 hours	
Thrombocytopaenia (platelets< 75x109 /l)			
Uncontrolled systolic hypertension (230/120 mmHg or higher)			
Untreated inherited bleeding disorders (such as hemophilia and von Willebrand’s disease)			

Data collection

The audit team comprised house officers, a general surgery registrar, a pharmacist, and a consultant. Data was collected prospectively from patients' medical notes, including information on age, sex, type of operation, whether VTE and bleeding risk assessments were performed, whether pharmacological or mechanical prophylaxis was administered, and any contraindications to VTE prophylaxis. Contraindications were also assessed by directly interviewing the patients. If the medical staff had not conducted VTE and bleeding risk assessments, an independent clinician, unaware of the audit, performed the assessment.

Additionally, we collected information about the type, dose, and duration of pharmacological prophylaxis and the time of applying mechanical prophylaxis. All data was anonymized and collected using an online Google form.

The total duration of this study was five months. Data collection lasted from 10/07/2022 to 10/09/2022 in cycle one and from 25/10/2022 to 20/12/2022 in cycle two. The results of cycles one and two were presented in local discharge meetings held by the surgical department on 17/09/2022 and 28/12/2022, respectively.

Action Plan

Educational Sessions

After completing cycle one and before starting cycle two, weekly educational sessions and focused group discussions were conducted from 20/09/2022 to 18/10/2022 in the general surgery department. These sessions were organized by the audit team in liaison with the surgery department. Each session lasted for one hour and a half to ensure the participation of all doctors (registrars, medical officers, and house officers) practicing in the general surgical department. These sessions focused on assessing VTE and bleeding risks, prescribing appropriate prophylaxis, and applying mechanical prophylaxis. The medical staff was also trained on how to use the risk assessment tool, which was later attached to each patient’s file. In addition, these sessions become part of the hospital induction sessions for the new doctors to ensure compliance with the guidelines.

Posters

After cycle one, posters demonstrating the summary of the NICE guidelines and Caprini score were distributed and made available in the hospital.

Statistical analysis

Data regarding VTE and bleeding risks assessment and prophylaxis was exported to an Excel sheet and was analyzed using SPSS software version 26 (SPSS Statistics (Version 27)). Continuous variables, including age, were reported as mean±standard deviation (SD). The frequency of each VTE risk was presented as a percentage of the total number of patients, and the percentages of adherence to the guidelines were recorded. Fisher’s exact test was used to test statistically significant differences in terms of correct practice between cycles one and two, and the two-sided p-value was measured with a 0.05 or less significance level.

## Results

In cycle one, 32 patients were included; 20/32 (62.5%) were females, 12/32 (37.5%) were males, and their mean age was 55.4±11.85 (mean±SD). In cycle two, 29 patients were included; 14/29 (48.3%) were females, and 15/29 (51.7%) were males, with a mean age of 53.48±10.70 (mean±SD). All those patients underwent different gastrointestinal operations, as listed in Table 3.

**Table 3:** Type of the operations

Operation	Cycle one (n=32)	Cycle two (n=29)
Cholecystectomy	12 (37.5%)	13 (44.8%)
Hernioplasty	7 (21.8%)	7 (24.1%)
Herniotomy	3 (9.40%)	0 (0.00%)
Subtotal gastrectomy	3 (9.40%)	1(3.4%)
Resection and primary anastomosis of small bowel	2 (6.3%)	3 (10.3%)
Loop ileostomy	2 (6.3%)	0 (0.00%)
Loop colostomy	2 (6.3%)	3 (10.3%)
Splenectomy	1 (3.1%)	0 (0.00%)
Reversed ileostomy	(0 0.00%)	2 (6.9%)

**Figure 1:**
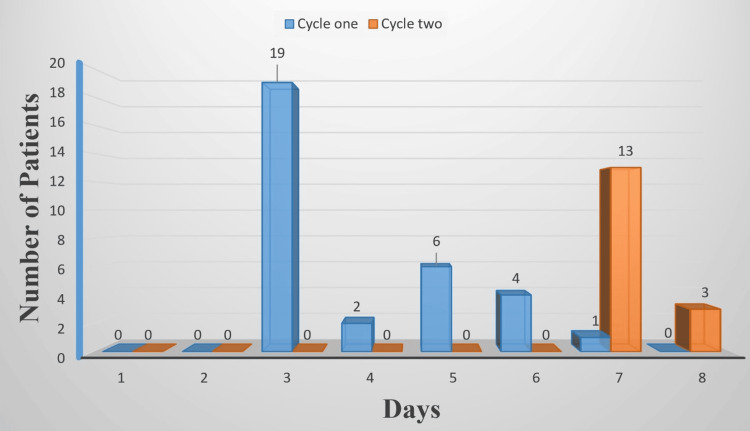
Duration of pharmacological prophylaxis in the two cycles: Number of patients who received pharmacological prophylaxis is 32 in cycle one and 16 in cycle two.

In cycle one, risk assessment for VTE and bleeding was done in 0/32 (0.00%) of patients. Pharmacological prophylaxis was given in 32/32 (100%) of those patients regardless of their VTE risk in the form of LMWH (5000 IU), and 0/32 (0.00%) of them received mechanical prophylaxis. All pharmacological prophylaxis was started postoperatively and stopped on discharge. The duration of pharmacological prophylaxis is summarized in Figure 1. 

 After performing VTE and bleeding risk assessments by the independent clinician, 18/32 (56.3%) of the patients were at high risk for VTE, and 14/32 (43.7%) were at moderate risk. None of these patients had any contraindications to mechanical or pharmacological prophylaxis.

In cycle two, risk assessment for VTE and bleeding was done in 29/29 (100%) of the patients. Of these, 16/29 (55.2%) were classified as high risk for VTE, and 13/29 (44.8%) were at moderate risk. All high-risk patients 16/16 (100%) received pharmacological prophylaxis postoperatively in the form of LMWH (5000 IU), however, only 10/16 (62.5%) of them also received mechanical prophylaxis in the form of anti-embolism stockings on admission. See Figure 1 for the duration of pharmacological prophylaxis. Among the moderate-risk patients, 7/13 (53.8%) received mechanical prophylaxis (anti-embolism stockings on admission) alone. None of the patients in cycle two had contraindications to medical or mechanical prophylaxis.

In cycle one, there was a gap between the standards and the practice. However, the results of cycle two showed a remarkable improvement in the practice, and the difference between cycle one and cycle two was statistically significant (p-value less than 0.05) (Table 4).

**Table 4:** Comparison of the practice of VTE prophylaxis prescription to the NICE standards The level of significance was calculated using the Fisher's Exact Test. The last row (Total) represents the sum of the high and moderate-risk groups in each cycle, allowing us to calculate the percentages of overall practice.

Risk	Cycle One (n =32)	Cycle two (n =29)	Standard	p-value
High	0/18 (0.00%)	10/16 (62.5%)	All patients should receive medical and pharmacological prophylaxis unless there is a contraindication	0.00006
Moderate	0/14 (0.00%)	7/13 (53.8%)	All patients should receive mechanical prophylaxis unless there is a contraindication	0.00104
Total	0/32 (0.00%)	17/29 (58.6%)	-	0.00001

## Discussion

Our study revealed significant gaps in the practice of VTE risk assessment and prophylaxis in the gastrointestinal surgery department at Alnao Teaching Hospital, Sudan. Cycle one showed poor compliance in adhering to the recommendation of VTE risk assessment according to the NICE guidelines, as 0/32 (0.00%) of patients received VTE and bleeding risk assessments. This issue likely results from a lack of awareness or training regarding the guidelines and risk assessment tools, such as the Caprini score. Despite this, postoperative pharmacological prophylaxis was given to all patients in cycle one 32/32 (100%) in the same way, without considering their unique risk profile. Moreover, during cycle one, mechanical prophylaxis was not used at all. A study done by Abdalla et al., in Alshuhada Teaching Hospital, Sudan, along with other studies conducted in low-income countries, showed similar results, highlighting the need for education on VTE prevention in low-resource settings like Sudan and emphasizing that the cause of non-adherence during the pre-intervention phase is the lack of sufficient understanding of the scope and burden of VTE [12,14,19,20].

The primary strategies for preventing VTE are pharmacological and mechanical prophylaxis, and it is essential to apply both techniques appropriately in order to lower the morbidity and mortality associated with VTE. Pharmacological prophylaxis (usually low molecular weight heparin, LMWH) was administered uniformly in both cycles of the current study; however, mechanical prophylaxis was generally underutilized in cycle one, with considerable improvements in cycle two. Similar studies from developing countries highlighted the gaps in thromboprophylaxis administration, with many patients at risk of VTE not receiving proper treatment due to prescription failures, fear of bleeding, or lack of knowledge and adherence to guidelines [12,13,20]. Underutilization of VTE prophylaxis is a widespread problem in developing countries, where clinical staff may not have the required education or awareness regarding the guidelines. Furthermore, the high cost of thromboprophylaxis, particularly mechanical measures, makes them inaccessible [21].

Cycle two showed a significant improvement after conducting the action plan. Risk assessment was performed for all patients 29/29 (100%), and the prescription of the recommended VTE prophylaxis based on patient risk increased substantially from 0/32 (0.00%) in cycle one to 17/29 (58.6%) in cycle two. Moreover, pharmacological prophylaxis was provided to all high-risk patients after excluding any contraindications, with a significant portion also receiving mechanical prophylaxis. However, there are still gaps since some moderate-risk patients were not given mechanical prophylaxis, and not all high-risk patients received the combination of pharmacological and mechanical prophylaxis.

In cycle one, only 1/32 (3%) of patients received low-dose LMWH for the recommended duration. This is likely due to the medical staff discontinuing LMWH upon discharge. This suggests a critical gap in the continuity of care, where the importance of maintaining VTE prophylaxis post-discharge was either not emphasized or overlooked. The improper duration of prophylaxis exposes high-risk patients to an increased chance of developing VTE and results in preventable morbidity or mortality [8,10]. In cycle two, all high-risk patients were given low-dose LMWH for the recommended duration. This significant improvement suggests that the educational sessions were effective in raising awareness among medical staff about the importance of adhering to VTE prophylaxis guidelines. These sessions clarified the necessity of continuing LMWH beyond discharge for the proper duration in high-risk patients, as specified by NICE guidelines. Education may also provide staff with practical knowledge on integrating these recommendations into discharge planning [8,20,22].

Due to the advanced healthcare infrastructure, better access to medical resources, and consistent adherence to clinical guidelines, VTE risk assessment procedures are more standardized and often widely implemented in developed countries. Studies from the United Kingdom, Australia, and Saudi Arabia showed more than 80% adherence to the guidelines [23-25]. On the other hand, developing countries frequently face challenges such as limited resources, lack of training, and inconsistent application of guidelines, leading to reduced levels of VTE risk assessment and prophylactic adherence. These differences show that in order to bridge the gap in VTE management, ongoing efforts in instruction, resource distribution, and guideline implementation in emerging contexts are required [12,13,15].

Several studies have highlighted barriers to effective VTE prevention, including inadequate protocol adherence, resource constraints, and the complexity of patient needs. Initiatives focused on quality improvement (QI), like multimodal interventions with computerized reminders and clinical alerts, have demonstrated promise in raising the rates at which thromboprophylaxis prescriptions are written. However, these interventions' effectiveness varies based on the hospital's context and healthcare infrastructure [7,26,27].

Educational interventions have proven to be effective in improving compliance with VTE prophylaxis guidelines [22,28]. The comparatively poor prophylactic recommendations compliance in the Egyptian arm in a study by Goubran et al suggests that healthcare providers need ongoing education [29]. Studies conducted in other developing nations, including Nigeria, have demonstrated that teaching programs greatly enhance the proper prescription of VTE prophylaxis [13]. However, the challenge remains to sustain these improvements over time. In developed countries, continual education, routine audits, and the integration of VTE risk assessment tools into standard clinical workflows are frequently used to ensure the long-term sustainability of improved practices. For instance, a UK study mentioned that Incorporating VTE prophylaxis techniques in hospital induction training for newly licensed physicians contributed to the long-term maintenance of high compliance rates [30]. Implementing similar continuous educational strategies in Sudan could lead to better adherence to VTE guidelines and more consistent patient outcomes.

Limitations

The limitations of this study include a small sample size, the focus on the gastrointestinal unit due to limited manpower and resources, and the lack of long-term follow-up to assess patient outcomes. In order to evaluate the long-term advantages of VTE prevention and find any gaps in post-hospital care, future research in Sudan and other developing countries should focus on following patients after they are discharged from the hospital. This will provide a more comprehensive understanding of the effectiveness of current prophylaxis strategies and highlight areas for further improvement.

## Conclusions

The audit emphasizes the importance of organized methods and ongoing education to improve adherence to VTE prophylactic recommendations and guidelines. The significant improvement between the audit cycles demonstrates that targeted interventions such as educational sessions, posters, and the use of risk assessment tools can lead to better practice, especially in low-resource settings. Continuous auditing and training are recommended to sustain and further improve adherence to VTE prophylaxis standards and guidelines in the surgical departments.
